# Reframing “One Health” for Healthcare-Associated AMR: Target Microbes, Internal Environments, and Operational Action

**DOI:** 10.3390/microorganisms14102366

**Published:** 2026-10-15

**Authors:** Ulf R. Dahle, Belinda Nicolau, Fernanda C. Petersen

**Affiliations:** 1 Division of Infection Control, Norwegian Institute of Public Health, P.O. Box 222, 0213 Oslo, Norway; 2 Faculty of Dental Medicine and Oral Health Sciences, McGill University, Montreal, QC H3A 0G4, Canada; belinda.nicolau@mcgill.ca; 3 Department of Oral Biology, Faculty of Dentistry, University of Oslo, 0316 Oslo, Norway; f.c.petersen@odont.uio.no

**Keywords:** antimicrobial resistance (AMR), One Health governance, healthcare-associated infections (HAI), hospital plumbing reservoirs, wastewater-based epidemiology (WBE), high-risk clones, Urban Wastewater Treatment Directive

## Abstract

While the One Health paradigm has successfully elevated antimicrobial resistance (AMR) on the global political agenda, its operationalization raises questions about how research, surveillance and interventions can best address transmission in healthcare settings. In this perspective, we argue for greater emphasis on four priorities in the application of One Health to healthcare-associated AMR. First, within the environmental dimension of One Health, internal hospital physical infrastructure, including wastewater plumbing, sink traps, and hospital drainage biofilms, warrants explicit attention because it can support the persistence, selection and transmission of resistant organisms. Second, as Article 17 of the recast Urban Wastewater Treatment Directive (Directive (EU) 2024/3019) establishes requirements for AMR monitoring in wastewater treatment plants serving agglomerations of 100,000 population equivalents and above, surveillance frameworks must match their analytical resolution to the intended public health objective. To bridge the current “actionability gap,” monitoring should combine population-level indicators with targeted genomic investigation of specialized epidemic clones and high-consequence mobile genetic elements where this detail can inform investigation or intervention. Third, global AMR strategies must balance targeted containment in clinical niches with guaranteed equitable access to essential, life-saving antimicrobials, particularly in low- and middle-income countries (LMICs) where inadequate access to effective treatment remains a major concern. Fourth, funding and evaluation criteria should judge One Health research by the importance of the question, the strength of the design and its relevance to prevention, rather than by the number of sectors represented, so that focused clinical and infrastructure studies are recognized as contributions to the wider agenda. Aligning regulatory mandates, funding mechanisms, and environmental surveillance with clinical and clonal realities is essential to strengthen the contribution of One Health to effective prevention and patient care.

## 1. Introduction: From Broad One Health Advocacy to Healthcare Realities

High-level advocacy and policy declarations have rightly warned that failure to confront antimicrobial resistance (AMR) risks plunging modern medicine into a post-antibiotic era where routine interventions become life-threatening [1]. These concerns have helped mobilize global attention to AMR, with One Health providing a framework for coordinated responses across human, animal and environmental health. Its implementation within healthcare raises practical questions about where preventive efforts should be directed.

High-consequence AMR poses particular risks within specialized, highly controlled human environments, such as neonatal intensive care units (NICUs), adult intensive care units (ICUs), oncology wards, and long-term care facilities. For a premature infant in a NICU, this controlled indoor setting represents a critical exposure pathway during a highly vulnerable period of life. While these settings remain connected to the wider community through patients, staff, caregivers, and materials, essential opportunities for prevention frequently lie close to the patient. Recent metagenomic evidence demonstrates that early antibiotic exposure and prolonged hospitalization in these units can profoundly shape the developing nasopharyngeal resistome, driving transient increases in resistance gene abundance and leaving long-lasting signatures of nosocomial pathogens such as Serratia marcescens [2]. To mitigate these trajectories, emerging prophylactic and microbiome-based paradigms, drawing parallels from mucosal interventions, offer potential complementary avenues for infection prevention [3]. Consequently, a balanced One Health framework should adequately account for these localized healthcare environments alongside broader ecological factors. To effectively combat AMR, the application of One Health should give explicit attention to localized healthcare environments. This can support commitments to reduce deaths associated with bacterial AMR by 10% by 2030 relative to 2019, ensure equitable access and prudent use of antimicrobials, and strengthen infection prevention and control (IPC) in healthcare services [4].

We have previously argued that the way One Health is operationalized by funders and regulators can favour cross-sectoral breadth over focused clinical and infrastructure research [5]. In our view, the application of One Health to healthcare-associated AMR should instead be guided by microbial ecology and the transmission pathways most relevant to patient risk, with research and surveillance priorities linked to opportunities for preventing infections caused by resistant pathogens and pursued alongside equitable access to effective antimicrobial treatment. This paper develops that position in three areas: the hospital built environment as part of the environmental dimension of One Health, the resolution needed to make wastewater surveillance actionable under the recast Urban Wastewater Treatment Directive [6], and the balance between containment and access. Specifically, national research councils and international funding bodies should apply these criteria during grant evaluation by prioritizing project proposals based on the scientific importance of the clinical question, the robustness of the epidemiological design, and the direct relevance to infection prevention, rather than enforcing rigid cross-sectoral participation quotas.

## 2. Aligning One Health Policies with Clinical AMR Realities

International strategies and political declarations on AMR have played an essential role in elevating AMR on the global agenda. By establishing the One Health paradigm as a core governance principle, international bodies have rightly emphasized that human, animal, and environmental health are intrinsically linked. Agricultural antibiotic use, zoonotic spillover, and environmental contamination contribute to the global resistome dynamics. The relative contribution of these pathways nevertheless varies by organism and setting, and research priorities should reflect that variation [7].

Furthermore, wastewater-based surveillance depends on centralized municipal collection infrastructure and treatment facilities, rendering it unviable in non-sewered rural areas and many LMIC settings. At the same time, any comprehensive One Health framework must account for human hosts, such as patients, healthcare personnel, and visitors, as the primary vectors introducing community-acquired pathogens into institutional environments.

The breadth of One Health is valuable because it encourages attention to connections that disciplinary boundaries can obscure. Yet a shared framework does not imply that every component of AMR has the same origin or requires the same combination of interventions. A resistant organism acquired during hospital care may be connected to wider reservoirs through its evolutionary history without those reservoirs being the most effective immediate targets for preventing transmission. Conversely, an apparently local outbreak may require investigation beyond the facility when epidemiological evidence points to a shared external source. The challenge is to retain this wider perspective while identifying the scale at which action is most likely to benefit patients.

The burden of infection among critically ill patients illustrates the importance of prevention within healthcare facilities [8]. In these settings, transmission can involve specialized, hospital-adapted bacterial lineages [9,10] and reservoirs in internal physical infrastructure such as plumbing systems, sink traps, and biofilms [11,12] antimicrobial exposure may also contribute to selection, depending on the organism and conditions [13].

This has a practical implication for research funding. One Health portfolios should accommodate both studies across sectors and focused investigations within healthcare. The inclusion of additional sectors should follow the scientific question and the pathways under investigation. In our view, funding criteria should recognize the contribution of focused clinical and infrastructure research to wider One Health goals [5]. A challenge in current strategies is that while publications from non-clinical environments are plentiful, translating and integrating these data into operational clinical administration and health policy frameworks can encounter systematic and administrative bottlenecks.

This is a question of how an overall research portfolio is assembled, rather than a requirement that each project encompasses the full One Health agenda. Focused studies can establish mechanisms or evaluate interventions that subsequently inform broader collaborations. Their value should depend on the importance of the question, the strength of the design and the relevance of the findings to prevention. Equally, a study spanning several sectors should make clear what their integration adds to the question being addressed. These principles allow scientific complementarity without treating the number of sectors represented as a substitute for likely public health benefit.

Priorities in LMICs must also reflect local healthcare and community conditions. In low-resource clinical settings, prevention of infections caused by resistant pathogens can be constrained by insufficient basic water, sanitation, and hygiene (WASH) infrastructure, severe hospital overcrowding, and a critical lack of dedicated infection prevention and control (IPC) resources within healthcare facilities [7]. In these vulnerable environments, high patient density combined with limited isolation capacity can accelerate the person-to-person and intra-facility transmission of resistant opportunistic pathogens. Building robust intra-hospital diagnostic capacity and local containment protocols should therefore accompany surveillance of relevant community and environmental pathways.

Global AMR strategies must also balance antimicrobial stewardship with equitable medicine access. Limited access, inappropriate use and resistance can coexist within the same health system. Laxminarayan and colleagues [14] highlighted the barriers that prevent patients from receiving effective antibiotics, including affordability, availability and weaknesses in health services.

In many LMICs, bacterial infections such as neonatal sepsis and pediatric pneumonia remain difficult to treat when effective antimicrobials are unavailable, unaffordable, or subject to fragile supply chains [14]. Efforts to reduce unnecessary antibiotic exposure should therefore be accompanied by measures to ensure timely treatment for patients who need it.

Access and prevention should also be considered together when choosing investments. A surveillance programme that identifies resistance but cannot support timely diagnosis, appropriate treatment or feasible infection control leaves a substantial part of the clinical problem unresolved. Where resources are limited, the case for an advanced analytical platform should include how its findings will reach the services responsible for acting on them. This does not diminish the importance of genomic capacity in resource-constrained settings. It places that capacity within a functioning pathway from recognition of risk to care, with resources for laboratory staff, clinical interpretation and sustained implementation.

To be ethically sound and clinically effective, global AMR strategies and funding structures must strike a dual balance:

- Targeted Containment: curbing unnecessary antibiotic exposure, industrial pollution, and transmission of high-risk epidemic clones in specialized clinical and environmental niches.
- Guaranteed Access: safeguarding the supply, affordability, and distribution of life-saving essential medicines to ensure that no patient dies from a treatable bacterial infection due to systemic supply failures [14].

## 3. Highlighting the Hospital Environment: Biofilms, Drains, and Infrastructure

Within the environmental dimension of One Health, the immediate physical environments of healthcare warrant explicit attention in research, surveillance and prevention. A substantial body of empirical literature established over the past decade documents resistance determinants in hospital sink drain biofilms [12]. Rather than representing novel scientific discoveries, addressing this persistent challenge often highlights the complex hurdles involved in translating existing environmental insights into routine clinical governance and facility design. Far from being passive, one-way waste conduits, internal plumbing networks harbor complex biofilm matrices that act as persistent microbial reservoirs. Antimicrobial residues have also been detected in clinical drainpipes [15]. Such exposure may create localized selective pressure, but its effects depend on concentrations, bioavailability and microbial community structure [13]. Detecting residues and resistance genes alone does not demonstrate ongoing selection or horizontal gene transfer.

Mechanistically, these physical niches can provide a link between the built infrastructure and vulnerable patients. In a model sink system, *Escherichia coli* embedded within drain biofilms was shown to grow upward toward sink strainers and disperse to nearby surfaces during faucet use [11]. For a critically ill or immunosuppressed patient in an ICU or NICU, this localized droplet dispersion represents a potential exposure pathway. Establishing transmission to a patient requires additional epidemiological and microbiological evidence.

Host-adapted microbial evolution is also relevant to these environments. Clinical environments frequently harbour epidemic hospital lineages, such as carbapenem-resistant *Klebsiella pneumoniae* [9] and hospital-adapted *Enterococcus faecium,* that can persist in infrastructure niches [10]. Furthermore, contemporary isolates of these pathogens can exhibit specialized phenotypic adaptations, such as increased alcohol tolerance under defined experimental conditions [16], highlighting how engineered clinical niches interact with microbial evolution (for detailed discussion of high-risk clones and lineage-specific transmission dynamics, see Section 5). This provides a more actionable operational focus for healthcare infection prevention data than broad environmental monitoring.

When resistant organisms enter the biofilm architecture of hospital plumbing, a persistent reservoir may develop. Patient carriage can seed the physical infrastructure, while contaminated infrastructure may contribute to subsequent patient exposure. Intra-hospital physical infrastructure therefore warrants explicit attention within One Health as a potentially important target for epidemiological research, funding, and infection prevention strategies.

Mathers et al. [17] reported reduced acquisition of KPC-producing organisms following an ICU wastewater intervention that included hopper covers. Although the observational design leaves questions about individual components and transferability, we consider such studies an important basis for developing this agenda. Infrastructure interventions should be assessed for their contribution to preventing patient acquisition and infection, alongside their effects on environmental reservoirs.

Giving hospital infrastructure greater operational attention also requires collaboration beyond the usual boundaries of clinical microbiology. Infection prevention teams, engineers, facilities managers and those responsible for purchasing and maintenance need a shared understanding of the exposure pathways under investigation. For example, a suspected sink-associated problem should prompt consideration of sink placement, drainage, patterns of use and proximity to patient-care materials, as well as microbiological findings. The appropriate response may involve changes to practice, maintenance or design, such as installing self-disinfecting thermal or heated sink traps, fitting splash barriers, and redesigning physical ward layouts to optimize the spatial separation of handwashing stations from patient-care zones, but these options should be evaluated in the setting concerned rather than assumed to be interchangeable.

Such resource-allocation considerations are particularly relevant in places where sanitation infrastructure and healthcare resources are constrained, broad environmental monitoring mandates risk diverting scarce funding and laboratory capacity away from fundamental IPC and basic hygiene infrastructure [7]. In these environments, focusing on feasible low-cost measures, such as basic hand hygiene compliance, pragmatic cohorting, and improved water safety, must take precedence. In LMIC settings, a risk-based prioritization framework may help focus environmental surveillance on high-risk transmission pathways, such as critical hospital drainage or municipal sanitation interfaces. Such targeted approach may ensure that environmental monitoring does not compromise frontline clinical diagnostics or basic IPC. A useful research agenda would distinguish the removal of detectable contamination from a sustained reduction in patient risk. Short follow-up may miss recolonization, while simultaneous changes in cleaning, screening and antibiotic use can complicate attribution of benefit. Comparative studies should therefore document these accompanying measures and consider maintenance requirements, unintended consequences and costs. A technically successful intervention that cannot be sustained by the facility may have limited preventive value. This emphasis on implementation connects the ecology of the built environment with the everyday conditions under which healthcare is delivered.

## 4. Connecting Wastewater Surveillance with Public Health Action: Governance, Methods, and the Recast UWWTD

The connection between hospital wastewater and municipal sewerage offers opportunities to link environmental observations with AMR prevention. However, a municipal signal does not need identify the source of a patient infection, and its interpretation depends on the sampling location and analytical approach. Comparative surveillance has shown that culture-based tracking of specific clinical pathogens, such as *Escherichia coli*, in municipal sewage and wastewater treatment plants can reflect regional resistance prevalence across the broader population [18]. However, a wastewater resistome profile cannot automatically be assumed to measure clinical infection prevalence. Different markers may inform population trends, environmental risks or resistance evolution, and should be selected and validated for the intended purpose [19].

Driven by recent European Union policy mandates, most notably Article 17 of the recast Urban Wastewater Treatment Directive (Directive (EU) 2024/3019) (UWWTD), wastewater-based epidemiology (WBE) for AMR is becoming a regulatory surveillance requirement in the EU for agglomerations of 100,000 population equivalents or more [6,20]. Implementation raises questions about methods, governance and the translation of findings into action [20].

Findings from the 2024 EU-Wastewater Integrated Surveillance for Public Health (EU-WISH) survey highlight this implementation gap: of 27 surveyed European countries, only 11 had active, operational AMR wastewater surveillance systems in place [20]. These systems display significant variance in focus, targeting extended-spectrum β-lactamase-producing *Enterobacterales* (ESBL-E), carbapenemase-producing *Enterobacterales* (CPE), vancomycin-resistant enterococci (VRE), and selected resistance genes. While culture-based methods dominate bacterial detection and quantitative PCR (qPCR) targets specific resistance genes, shotgun metagenomics is only occasionally deployed due to complex biological and bioinformatic interpretation [20].

A structural challenge is to define institutional responsibilities for routine sampling, testing, interpretation and response, including coordination among municipal water utilities, environmental protection agencies and national public health institutes [20]. Empowering specialized national reference laboratories within this architecture is helpful to provide centralized diagnostic consistency, high-resolution strain-typing, and technical oversight. Crucially, data generation needs to be linked to defined public health actions. When metagenomic screening or qPCR detects an elevated abundance of specific resistance genes in a municipal wastewater treatment plant serving hundreds of thousands of residents, the appropriate next steps for clinical decision-makers or local health authorities depend on the surveillance objective and corroborating evidence. Without clear intervention pathways, broad wastewater surveillance risks becoming a data collection exercise whose contribution to public health decisions is difficult to demonstrate. Furthermore, the detection of resistance determinants or organisms in raw sewage does not directly reflect clinical epidemiological risk. In the absence of standardized sampling protocols, uniform culture media, PCR primers, or nucleic acid extraction methods, and without regulatory limits on bacterial loads in treated effluent, raw wastewater monitoring can struggle to deliver directly comparable data across jurisdictions, thereby risking inefficient resource utilization.

Population-level trend monitoring and early warning can have value even when a signal does not identify an individual infection or its source. In our view, the value of surveillance should be judged by its contribution to decisions and prevention, including benefits that emerge over time. Existing frameworks and reviews provide a basis for this assessment, while identifying gaps in documentation of the benefits of integration [21,22].

This distinction matters, in particular, when results move between municipal surveillance and hospital infection control. A catchment-wide increase may justify closer observation or a regional investigation without identifying any particular facility as its source. Hospital effluent offers a more focused sampling frame, but still combines contributions from colonized patients, infected patients and organisms persisting in the plumbing. Neither sampling scale provides an uncomplicated measure of incident infection. Connecting the two could nevertheless help determine where additional investigation is justified, provided that sampling boundaries, patient populations and clinical data are considered together.

We consider the resources needed for follow-up to be part of the surveillance programme itself. Before introducing a marker, the responsible organizations should agree who will review an unexpected result, who can request confirmatory testing and who will decide whether further sampling or clinical investigation is warranted. These arrangements should include the ability to conclude that a signal does not require escalation. Otherwise, improved analytical sensitivity may generate repeated alerts without improving the allocation of preventive effort. A programme should also be able to assess missed events, since an apparently stable wastewater signal does not exclude a localized outbreak.

To bridge this “actionability gap,” surveillance programmes could align surveillance targets with specific monitoring objectives [20]. We propose the following possible responses for evaluation, with thresholds and responsibilities adapted to local conditions:

Targeted Upstream Auditing: Unexpected increases in high-priority carbapenemase genes should first prompt checks of analytical quality, flow conditions and consistency across samples. Persistent, corroborated signals may justify targeted sub-catchment sampling (e.g., hospital effluent lines or long-term care facilities) to investigate contributing sources.

Stewardship Realignment: Wastewater trends can contribute to regional stewardship assessments. Changes to prescription guidelines require corroborating clinical susceptibility data and information on the infections concerned.

Infection Control Investigation: Sustained detection of high-risk epidemic clones, supported by clinical or epidemiological information, can justify targeted screening, audits, and hygiene interventions in implicated healthcare facilities.

Infrastructure Investments: Consistently high emissions of viable multidrug-resistant pathogens or active antibiotic residues in treated effluent can inform evaluation of additional treatment technologies (e.g., ozonation, advanced oxidation, or membrane filtration) at specific wastewater treatment plants to protect receiving aquatic environments. Treatment performance could be evaluated separately for antibiotic residues, viable bacteria and resistance genes [6,20].

Furthermore, the lack of standardized, cross-border methodologies poses barriers to comparative analysis. Methodological heterogeneity persists across the analytical pipeline, from sampling techniques (e.g., passive samplers versus 24-h flow-proportional composite samples) and target selection (culture-based isolate recovery versus shotgun metagenomics or targeted qPCR) to bioinformatic pipelines and normalization metrics [20]. Environmental factors, such as stormwater run-off, variable flow rates, and industrial effluent, can dilute biological signals and introduce confounding variables that vary widely between geographic regions and seasons. Normalization of antibiotic resistance gene (ARG) copy numbers, including the use of fecal markers, requires validation for the setting and objective; no single denominator can be assumed to resolve all sources of variation [20].

Normalization of ARG copy numbers requires the establishment of site-specific baseline levels to account for the ubiquitous presence of resistant bacteria and ARGs in the modern antibiotic era. Additionally, normalization metrics and tracking targets must account for non-fecal environmental and skin-colonizing pathogens within the ESKAPE spectrum, rather than relying exclusively on fecal markers.

Collaborative initiatives such as the EU-WISH Joint Action, EU-JAMRAI 2, the WHO Tricycle protocol, and ISO Technical Committee 147 (SC4) are actively working toward harmonizing target selection, sampling schemes, and quality assurance [20]. Shared controls, transparent metadata and interlaboratory comparisons can improve comparability while methods and standards continue to develop. To strengthen wastewater monitoring as a One Health tool, policy-makers must align regulatory mandates with clear governance structures, standardized protocols, and defined mechanisms for translating environmental signals into concrete clinical and public health action [20].

To illustrate how such a decision framework could operate in clinical practice, we outline a four-step framework distinguishing between regional sewer tracking and targeted hospital-infrastructure investigations:

1. Signal detection and validation: Surveillance detects an emerging high-risk resistance determinant or epidemic clone. At the municipal level, this functions as a broad regional warning of community or healthcare-associated circulation, whereas at the intra-hospital level (e.g., specific building drainage), it points to localized institutional reservoirs.
2. Compartmental attribution: Epidemiological and genomic tracing assesses whether the detected signal originates from known hospital inpatients under active isolation, unrecognised silent carriage, or external community sources.
3. Targeted investigation and action: If the signal is tracked to internal hospital infrastructure (such as specific sink traps, branch drains, or plumbing networks), targeted environmental sampling and infection control measures, such as enhanced hygiene auditing, localized disinfection, or pragmatic cohorting, are deployed. If the signal stems from municipal wastewater, investigations focus on regional clinical and community interfaces rather than pipe-by-pipe tracking.
4. Outcome evaluation and sustainability: The impact of the intervention is evaluated by monitoring subsequent wastewater signal loads alongside clinical surveillance metrics, ensuring that measures are sustainable and do not divert resources away from core frontline diagnostics.

## 5. Microbe-Specific One Health: Resolving High-Risk Clones Within Broader Surveillance

Whether evaluating intra-hospital transmission or interpreting municipal wastewater signals under regulatory mandates, the microbial resolution needed depends on the question and the decisions to be supported. Broad measures of “AMR in the environment” can obscure important distinctions because different bacterial species, and even distinct lineages within the same species, operate through fundamentally different ecological, evolutionary, and transmission rules. Foodborne zoonotic pathogens, including many *Campylobacter* and *Salmonella* populations, illustrate cross-sectoral transmission routes connecting livestock, food systems, wildlife reservoirs, and human populations [23]. Conversely, obligate or predominantly human pathogens, such as *Neisseria gonorrhoeae* or *Mycobacterium tuberculosis*, rely primarily on direct human to human transmission [24,25]. Consequently, their surveillance and management rely on clinical diagnostics, patient treatment registries, and contact-tracing strategies rather than environmental or wastewater tracking. Host range alone does not determine the value of environmental sampling; that value depends on the objective and the performance of the method.

Particular analytical and operational challenges arise within the opportunistic spectrum (*Escherichia coli*, *Klebsiella pneumoniae*, *Enterococcus faecium*). Species-level surveillance metrics can obscure lineage-specific contributions to clinical resistance. Effective stewardship, resource allocation, and research funding can benefit from targeted analysis of high-risk clones and mobile genetic elements (MGEs) alongside species-level measures. For example, the global dissemination of carbapenem-resistant *K. pneumoniae* involves specific epidemic lineages (e.g., ST11, ST258, ST147, ST307) associated with healthcare transmission [9,26]. Similarly, *Enterococcus faecium* clade A1 represents a specialized, hospital-adapted lineage with a distinctive evolutionary history and a strong association with healthcare-associated infections (HAIs) [10]. Increased alcohol tolerance in hospital *E. faecium* isolates provides an example of adaptation under defined experimental conditions [16]. This observation should not be interpreted as evidence that correctly applied alcohol-based hand hygiene is ineffective. Lineage membership alone, however, does not establish an exclusively hospital reservoir or a recent transmission event.

Norwegian genomic data illustrate the importance of this distinction. Hetland et al. [27] found overlapping *K. pneumoniae* species-complex populations across reservoirs, but relatively few recent transmission events between ecological niches. Such findings support context-specific priorities while retaining the capacity to investigate exchange across reservoirs. Sampling design and the movement of mobile resistance determinants must also be considered [28].

The relevant unit of investigation may therefore change as an event unfolds. A rise in resistance within one species might lead to investigation of an expanding lineage; detection of the same resistance mechanism in several species might instead raise questions about a mobile element or a shared source. These are hypotheses to be tested with genomic and epidemiological information. Sequence type alone is too coarse to establish a recent transmission link, while a resistance gene detected without its host context may have several possible explanations. The value of additional resolution lies in distinguishing these explanations sufficiently to guide the next decision.

This approach also avoids restricting attention to a fixed list of familiar epidemic clones. Established lineages are useful targets where they account for an important local burden, but surveillance must retain the capacity to recognize emerging lineages and new combinations of resistance and host background. Targeted recovery and sequencing can be combined with broader approaches for this purpose. The balance should be revisited as the local epidemiology changes, rather than allowing a technically convenient marker panel to define the problem indefinitely.

The value of clonal granularity should inform the implementation of wastewater monitoring under Article 17 of the recast Directive. Shotgun metagenomics or targeted quantitative PCR (qPCR) of bulk municipal influent can identify resistance markers, but gene abundance alone may not identify the bacterial host or establish viability. Low-abundance clinical lineages may require targeted recovery and sequencing [20]. To make wastewater surveillance actionable for hospital infection control, targeted upstream sampling may be needed to distinguish community contributions from hospital effluent. High-risk clones carried by patients can enter municipal sewerage. Detecting an increase at targeted sub-catchments or hospital discharge pipes may support IPC investigations when interpreted alongside clinical and epidemiological evidence. Broad trend monitoring should therefore be combined with more detailed genomic analysis when a signal warrants investigation.

Furthermore, gaps in wastewater treatment infrastructure can allow untreated hospital effluent to mix directly with urban and agricultural water bodies, increasing environmental exposure to multidrug-resistant pathogens [13]. In these open aquatic environments, resistant organisms may encounter pharmaceuticals and heavy metals that can contribute to co-selection under suitable conditions. The extent of selection and gene exchange requires investigation in each setting. Distinguishing between genuine zoonotic spillover, environmental gene amplification, and the dissemination of specialized healthcare-associated clones is therefore essential. Surveillance frameworks should provide access to clonal and mobile-element resolution where it can clarify these processes or guide containment, while maintaining feasible baseline monitoring. Rather than conflicting with the broader monitoring requirements set forth in frameworks such as Article 17, a risk-based prioritization approach ensures that these extensive datasets are effectively translated into actionable local interventions without overwhelming laboratory capacity or diverting resources from frontline diagnostics.

## 6. Conclusions and Strategic Call to Action

To strengthen the application of One Health to healthcare-associated AMR, researchers and policy-makers should align activities with their intended purpose and local transmission pathways. Broad monitoring and targeted investigations serve complementary purposes and should be connected to defined decisions. We propose four priorities for strengthening the contribution of One Health to the prevention of healthcare-associated AMR:

Give Explicit Attention to Hospital Environments: Research and prevention programmes should give explicit attention to the indoor hospital micro-environment, including wastewater plumbing, sinks, biofilm reservoirs, and medical devices, within the environmental dimension of One Health. Investigating these engineered clinical niches and evaluating interventions can help translate recognition of environmental reservoirs into improved IPC [11,17].

Prioritize High-Resolution Microbial and Genomic Granularity: National surveillance programs, environmental sampling initiatives, and regulatory frameworks should complement species-level counts and resistance gene abundance with targeted investigation of high-risk epidemic clones and mobile genetic elements (MGEs). As the operational challenges of Article 17 illustrate, the public health value of surveillance depends on its objectives, interpretation and links to action [20]. Where the investigation requires it, surveillance should resolve specialized healthcare-associated lineages (e.g., ST258/ST307 *K. pneumoniae* [26] or *E. faecium* clade A1) and high-consequence resistance mechanisms to enable targeted upstream audits and rapid public health interventions.

Align Research Priorities with Clinical Reality: The scope of research in specialized, high-vulnerability units, such as NICUs, oncology wards, and long-term care facilities, should follow the scientific question and relevant exposure pathways. Protecting high-risk patients requires appropriate collaboration across clinical, microbiological and facilities expertise, and effective IPC. These goals can contribute to wider One Health priorities.

Judge Research by Question and Design, Not Sectoral Breadth: Funding and evaluation criteria for One Health research should recognize focused clinical and infrastructure studies as contributions to the wider agenda. The value of a proposal should rest on the importance of the question, the strength of the design and the relevance of the findings to prevention, rather than on the number of sectors represented [5]. Studies that span several sectors should likewise state what their integration adds. Portfolios assembled on this basis can accommodate both broad cross-sectoral work and targeted investigation within healthcare, and should be paired with sustained commitments to equitable access to diagnostics and effective antimicrobials, so that containment efforts do not leave treatable infections untreated [14].

Translating high-level political commitments into measurable reductions in infections caused by resistant pathogens requires clear links between evidence, decisions and interventions. By aligning regulatory mandates, funding mechanisms, and environmental surveillance with the specific transmission dynamics of high-risk lineages, policy-makers can strengthen One Health as an actionable, life-saving framework for global health security.

## Data Availability

No new data were created or analyzed in this study. Data sharing is not applicable to this article.
